# Exploring the potential for a new measure of socioeconomic deprivation status to monitor health inequality

**DOI:** 10.1186/s12939-022-01661-0

**Published:** 2022-04-23

**Authors:** Jakob Dirksen, Monica Pinilla-Roncancio, Fernando C. Wehrmeister, Leonardo Z. Ferreira, Luis Paulo Vidaletti, Katherine Kirkby, Theadora Swift Koller, Anne Schlotheuber, Heriberto Tapia, Cecilia Vidal Fuertes, Sabina Alkire, Aluisio J. D. Barros, Ahmad Reza Hosseinpoor

**Affiliations:** 1 Oxford Poverty and Human Development Initiative, University of Oxford, Oxford, UK; 2 Universidad de los Andes, Bogotá, Colombia; 3 International Center for Equity in Health, Post-Graduate Program in Epidemiology, Federal University of Pelotas, Pelotas, Brazil; 4 World Health Organization, Geneva, Switzerland; 5 Human Development Report Office, United Nations Development Programme, New York, USA

**Keywords:** Health inequalities, RMNCH, Global health, Equity, Disadvantage, Measurement, Monitoring inequalities, Data disaggregation, Socioeconomic deprivation

## Abstract

**Background:**

Monitoring health inequalities is an important task for health research and policy, to uncover who is being left behind – and where – and to inform effective and equitable policies and programmes to tackle existing inequities. The choice of which measure to use to monitor and analyse health inequalities is thereby not trivial. This article explores a new measure of socioeconomic deprivation status (SDS) to monitor health inequalities.

**Methods:**

The SDS measure was constructed using the Alkire-Foster method. It includes eight indicators across two equally weighted dimensions (education and living standards) and specifies a four-level gradient of socioeconomic deprivation at the household-level. We conducted four exercises to examine the value-added of the proposed SDS measure, using Demographic and Health Surveys data. First, we examined the discriminatory power of the new measure when applied to outcomes in four select reproductive, maternal, neonatal, and child health (RMNCH) indicators across six countries: skilled birth attendance, stunting, U5MR, and DTP3 immunisation. Then, we analysed the behaviour and association of the new SDS measure vis-à-vis the DHS Wealth Index, including chi-squared test and Pearson correlation coefficient. Third, we analysed the robustness of the SDS measure results to changes in its structure, using pairwise comparisons and Kendal Tau-b rank correlation. Finally, we illustrated some of the advantageous properties of the new measure, disaggregation and decomposition, on Haitian data.

**Results:**

1) Higher levels of socioeconomic deprivation are generally consistently associated with lower levels of achievements in the RMNCH indicators across countries. 2) 87% of all pairwise rank comparisons across a range of SDS measure structures were robust. 3) SDS and DHS Wealth Index are associated, but with considerable cross-country variation, highlighting their complementarity. 4) Haitian households in rural areas experienced, on average, more severe socioeconomic deprivation as well as lower levels of RMNCH achievement than urban households.

**Conclusions:**

The proposed SDS measure adds analytical possibilities to the health inequality monitoring literature, in line with ethically and conceptually well-founded notions of absolute, multidimensional disadvantage. In addition, it allows for breakdown by its dimensions and components, which may facilitate nuanced analyses of health inequality, its correlates, and determinants.

**Supplementary Information:**

The online version contains supplementary material available at 10.1186/s12939-022-01661-0.

## Background

Health outcomes, opportunities, and interventions are unequally distributed worldwide and vary both within and across countries. Monitoring health inequalities is an important task for health research and policy, to uncover who is adversely affected – and where – and to inform effective and equitable policies and programmes to tackle existing inequities.

The process of monitoring health inequalities includes the selection of suitable dimensions of inequality, which can constitute a source of discrimination associated with different health outcomes [1]. Many dimensions of inequality exist. Aspects related to socioeconomic (e.g. household or individual wealth, income, occupation, expenditure and education), demographic (e.g., sex and age), and geographic characteristics (e.g., urban–rural area or subnational region) have been identified as important for the analysis of health inequalities globally [2], amongst other stratifiers such as ethnicity, migrant status, religion, caste, etc. The selection of which dimension to use depends on the context, the population, and the purpose of monitoring.

The health inequality monitoring literature makes frequent use of the DHS Wealth Index to identify socioeconomic position. This index relies on a list of assets and goods to indicate households’ relative position in terms of material living standard [3]. One limitation of the Wealth Index is that it is insensitive to the possibly considerable variation of absolute (dis)advantage among worst-off groups – bottom wealth quintiles, for example – across countries and over time. The wealth index may thus be useful for one-off assessments of within-country inequalities, but has limited use for cross-country comparisons and intertemporal analyses [4]. Further exploration into approaches for health inequality monitoring that address this and other limitations is timely. See also [5, 6] for additional limitations of the DHS Wealth Index and some proposals for their mitigation.

This article explores a new measure of Socioeconomic Deprivation Status (SDS) to support global health inequality monitoring efforts. The proposed measure aims to contribute to an enhanced understanding of the associations between health indicators and socioeconomic deprivations related to living standards and education. Compared to other commonly used measures, it has several properties that are advantageous for health inequality monitoring and comparative as well as aggregated and disaggregated analyses across time and space. Furthermore, the SDS measure directly operationalises the widespread notion that disadvantage is multidimensional by capturing joint and intersecting deprivations at the household level (e.g. [7]). It is also aligned with understanding disadvantage as an absolute, rather than a relative, phenomenon (e.g., [8, 9]).

This article introduces the motivation for, and the specific structure of, the new SDS measure and then illustratively assesses its discriminatory power for health inequality monitoring across six countries and four RMNCH indicators, using recent Demographic and Health Surveys (DHS). The article also presents results of sensitivity and robustness analyses for the new SDS measure, based on the most recent DHS data for a set of 46 countries. It empirically illustrates some of its advantages, as well as limitations and weaknesses, vis-à-vis other measures that are and have been used for health inequality monitoring around the world. Finally, it suggests next steps in further testing/piloting the index.

## Methods

### The Alkire-Foster method

The SDS measure is constructed using the Alkire-Foster method [10, 11]. This method axiomatically defines a class of multidimensional measures, allowing for a variety of parametric choices including (relevant for the present context) the selection of binary deprivation/non-deprivation indicators, their relative weights, the specification of deprivation cutoffs (i.e., the point at which a certain deprivation/non-deprivation applies), and overall multidimensional cutoffs.

As part of any measure constructed using the Alkire-Foster method – including the SDS – a deprivation profile is constructed for each unit of identification (here: households). Based on specific deprivation cutoffs, a household and all its members are first identified as either deprived or non-deprived in each indicator. These binary deprivation indicators are then multiplied by the respective weights that have been selected for each indicator. These weights reflect the importance of each indicator for overall socioeconomic deprivation. Each weighted deprivation profile is then summarised as an overall deprivation score and compared against one or more overall multidimensional cutoffs. Because of its two-cutoff procedure – individual deprivation cutoffs and overall multidimensional cutoff – the Alkire-Foster method is also referred to as the dual cutoff counting approach, ‘counting’ the weighted deprivations that households and all of their members experience. The second cutoff allows for the two classic steps of identification and aggregation in deprivation measurement*,* following Sen [12]. Identification considers whose weighted deprivation score is greater than or equal to any multidimensional cutoff. Aggregation then summarises the information on those who are poor according to the multidimensional cutoff into population-level measures.

The aggregate-level measures most commonly constructed with the Alkire-Foster method are the so-called adjusted headcount ratio or ${M}_{0}$ and its two partial indices $H$ and $A$. $H$ is the headcount ratio or incidence of multidimensional disadvantage (or socioeconomic deprivation). It expresses the percentage of people who are multidimensionally disadvantaged, given the chosen multidimensional cutoff. $A$ is the intensity and expresses the average share of weighted deprivations that multidimensionally disadvantaged individuals face. The ${M}_{0}$ is an adjusted headcount ratio because it is the multiplicative adjustment of the headcount ratio with the average intensity, i.e., the product of incidence and intensity ($H\times A$). It represents the deprivations that those multidimensionally disadvantaged experience (0–1), expressed as a percentage of the total possible deprivations, i.e., the deprivations that would be experienced if everyone in society was deprived in all indicators included in the measure in question.

The SDS measure relies on multiple multidimensional cutoffs to construct a gradient of multidimensional disadvantage identification functions and corresponding headcount ratios ($H$) for distinct ranges of weighted deprivation scores. For illustrative purposes, this article presents the further step of aggregation into an adjusted headcount ratio. This demonstrates two critical axioms of the Alkire-Foster class – subgroup decomposition and dimensional breakdown. As a multidimensional extension of the Foster-Greer-Thorbecke class of measures – the most widely applied class of measures for monetary poverty estimation – the Alkire-Foster class and its extensions do also comprise measures that are additionally distribution-sensitive with increasing prioritisation of the worst-off [10, 11]. Such applications are beyond the aims and scope of the present study, but may be insightful in future research, particularly where deprivational indicators are fully cardinal or cardinalised.

### The measure of socioeconomic deprivation status

The SDS measure comprises eight indicators across two dimensions: two indicators in the education dimension and six indicators in the living standards dimension (see Table 1 for details). Both dimensions are equally weighted at 50%, and indicators within each dimension receive equal weight, i.e., 25% each in the education dimension and ~ 8.3% each in the living standards dimension, so that possible weighted deprivation scores range from 0–1 in discrete intervals of 1/12. These specifications are inspired by, and closely follow the ones of, the global Multidimensional Poverty Index annually published by the United Nations Development Programme (UNDP). See e.g. [13]. Just as the global Multidimensional Poverty Index, the SDS measure is internationally comparable across more than 100 countries and combines two dimensions of socioeconomic status (living standards and education) that are widely recognised as core constituents of human development and deprivation [11, 13]. Furthermore, both measures can be readily estimated from Demographic Health Surveys (DHS) and Multiple Indicator Cluster Surveys (MICS) – two of the most important data sources for the monitoring and analysis of health outcomes, interventions, and inequalities around the globe. Different from the global Multidimensional Poverty Index, the SDS measure does not include a health dimension, because for health inequality monitoring that would effectively mean a disaggregation of health indicators by themselves. The unit of identification for single deprivation and multidimensional disadvantage is the household, whilst results can be reported and analysed at the individual level. Four intervals around three multidimensional disadvantage cutoffs are established at 25%, 50% and 75% of the weighted sum of deprivations, respectively. A cutoff of 25%, for example, implies that a household deprived in at least one education indicator or three living standards indicators or – for that matter – any combination of indicators whose weighted sum amounts to at least 25%, is identified as multidimensionally disadvantaged. The same logic applies to the values of 50% and 75%, respectively. This gradient is then used to identify people within each of these ranges of socioeconomic deprivation, creating four subgroups subsequently used to stratify health outcomes and interventions, i.e., 0%- < 25%; 25%—< 50%; 50%- < 75%; and 75%-100% of the weighted deprivations included in the SDS measure.

**Table 1:** Structure of the SDS measure: Dimensions, indicators, and weights

Dimensions of Disadvantage	Indicator	Deprived if…	Weight
Education	Years of schooling	**No** eligible household member has completed at least **six years** of **schooling**.^a^	1/4
	School attendance	Any school-aged child is **not attending** school **up to** the age at which they would complete **class 8**.^b^	1/4
Living Standards	Cooking fuel	A household cooks using **solid fuel**, such as dung, agricultural crop, shrubs, wood, charcoal, or coal	1/12
	Sanitation	The household has **unimproved** or **no** sanitation **facility** or it is improved but **shared** with other households.^c^	1/12
	Drinking water	The household’s source of **drinking water** is **not safe** or safe drinking water is a **30-min** or **longer walk** from home, roundtrip.^d^	1/12
	Electricity	The household has **no electricity**.^e^	1/12
	Housing	The household has **inadequate** housing materials in **any** of the three components: **floor**, **roof,** or **walls**.^f^	1/12
	Assets	The household does **not own more than one** of these **assets**: radio, TV, telephone, computer, animal cart, bicycle, motorbike, or refrigerator, and does not own a car or truck	1/12

Source: Based on [14]

^a^If all individuals in the household are in an age group where they should have formally completed 6 or more years of schooling, but none have this achievement, then the household is deprived. However, if any individuals aged 10 years and older reported 6 years or more of schooling, the household is not deprived. In the case that a household does not have eligible members the household is considered as non-deprived

^b^Data sources for the age children start compulsory primary school: DHS survey reports and http://data.uis.unesco.org/

^c^A household is considered to have access to improved sanitation if it has some type of flush toilet or latrine, or ventilated improved pit or composting toilet, provided that they are not shared

^d^A household has access to safe drinking water if the water source is any of the following types: piped water, public tap, borehole or pump, protected well, protected spring or rainwater, and it is within a 30-min walk, round trip

^e^A number of countries do not collect data on electricity because of 100% coverage. In such cases, we identify all households in the country as non-deprived in electricity

^f^Deprived if floor is made of natural materials or if dwelling has no roof or walls or if either the roof or walls are constructed using natural or rudimentary materials

## A first set of empirical applications

### Methods and data

We carried out four analytical exercises using the proposed measure of SDS. Below we discuss their results and implications for the appropriateness and value-added of the SDS measure for global health inequality monitoring efforts. The first exercise examined the discriminatory power of the new measure when applied to four select RMNCH indicators and compared coverage and prevalence rates of these RMCNH indicators by SDS. The second exercise analysed the behaviour of the new SDS measure vis-à-vis the DHS Wealth Index as measures for health inequality monitoring. The analysis uses a dependency test (chi-squared test) and a correlation between both measures to study their consistency. The third exercise analysed the sensitivity and robustness of the newly proposed SDS measure by applying parametric modifications to the measure, i.e. the multidimensional disadvantage cutoff, using data from 46 Demographic and Health Surveys (see Table A1). The third exercise followed common practice in the measurement literature (e.g. [11, 15]). The final and fourth exercise illustrated some of the axiomatic properties and advantages of the SDS measure. This exercise used Haiti DHS data with a single cutoff at 50% of the weighted SDS deprivations. It computed the incidence $(H)$, intensity $(A)$, and adjusted headcount ratio $({M}_{0})$ in order then to illustrate subgroup disaggregation and indicator-wise breakdown – here by rural and urban areas.

All empirical exercises used DHS data from nationally representative household surveys. For the first two exercises, data covered six countries spanning all six WHO regions: Haiti (2016), India (2015–16), Niger (2012), Pakistan (2017), Papua New Guinea (2016), and Tajikistan (2017). In the first exercise, the SDS measure was applied to the four RMNCH indicators: 1) Skilled birth attendance, measured as the proportion of live births to women aged 15–49 years that were attended by skilled health personnel in the period prior to the survey; 2) DTP3 immunisation, measured as the proportion of children aged 12–23 months having received three doses of the combined diphtheria, tetanus toxoid and pertussis (DTP3) vaccine in a given year; 3) Stunting, measured as the proportion of children aged less than five years who are stunted (defined as more than two standard deviations below the median age and sex-specific height-for-age of the WHO Child Growth Standards, i.e., z-score below minus 2 standard deviations (< -2 SD), indicating moderate or severe stunting) [16]; and 4) Under-five mortality, which is the probability (expressed as a rate per 1000 live births) of a child born during a ten-year period prior to the survey dying before reaching the age of five years [17]. For more details on these indicators, see [18]. The second exercise used the DHS Wealth Index [3]. The DHS Wealth Index relies on principal component analysis, a data reduction technique aimed at capturing maximal variation in a large set of interrelated variables through a few new variables – so-called principal components. The DHS Wealth Index is exclusively the first principal component of material living standards variables included in DHS, subsequently used for the construction of endogenously defined relative wealth quintiles and deciles [3]. We compared the association between the SDS measure and the DHS Wealth Index using a Chi2 test when using categorical variables and a Pearson correlation for continues variables. The third exercise used DHS data from 46 low- and middle-income countries collected between 2010-2018. For the final exercise, we used DHS Haiti (2016) data to illustrate useful properties of the SDS measure for health inequality monitoring and analyses.

## Results

This section presents some general results of the new SDS measure for the 6 countries for which data were analysed in exercises 1 and 2, followed by results of each of the four exercises described above.

### Multiple deprivation profiles in 6 countries

As the first result of initial exploration of data from Haiti (2016), India (2015–16), Niger (2012), Pakistan (2017), Papua New Guinea (2016), and Tajikistan (2017), findings revealed that the higher the multidimensional cutoff, the lower the percentage of the population whose deprivation load meets or exceeds it (see Table 2). This was expected. Take, for instance, the case of Haiti. When using a multidimensional cutoff equal to $25\%$, around 76.6% of the population was identified as multidimensionally disadvantaged. For a cutoff equal to $50\%$, this figure was 26.1%, and for a cutoff equal to $75\%$ it was 13.0%. Similar results were found for the remaining five countries. In Tajikistan, no one was identified as extremely deprived, i.e., none of those surveyed experienced a deprivation load equal to or greater than 75% of the weighted deprivations included in the SDS measure, and only 0.1% of the population experienced at least half of the weighted SDS deprivations. This provided an initial overview of multiple deprivation profiles in the six countries that were studied for illustrative purposes. The SDS measure as a gradient can then be expressed by subtracting the percentage of each interval from 100%. For example, since 42.6% of the population in India are deprived in at least 25% of the weighted SDS indicators, 57.4% of the population will here fall in the lowest 0% to < 25% intensity interval. 42.6%-9.8% (or 32.8%) are deprived in the 25% to < 50% interval, 9.8%-2.1% (or 7.7%) are deprived in the 50% to < 75% interval, and, finally, 2.1% are deprived in at least 75% of weighted SDS indicators. Findings reported in Table 2 also show that the relative size of the SDS gradient subgroups varies considerably across countries. In Niger, for example, the share of the population identified extremely deprived is, with 56.7%, considerably larger than any other country illustratively studied here. Results for each SDS intensity interval are a function of the distribution of joint SDS deprivations in each country. These may be affected by the levels of human, social and economic development. Therefore, it is not unsurprising that the populations of countries with higher levels of human development are subject to, on average, lower levels of socioeconomic deprivation.

**Table 2:** Percentage of the population that is disadvantaged, as per the proposed SDS measure, by different cut-off values in six selected countries

Country	Cutoff = 25%	Cutoff = 50%	Cutoff = 75%
Haiti	76.6%	26.1%	13.0%
India	42.6%	9.8%	2.1%
Niger	95.1%	80.1%	56.7%
Pakistan	46.8%	18.4%	8.7%
Papua New Guinea	88.6%	38.4%	25.0%
Tajikistan	13.5%	0.1%	0.0%

#### Exercise 1: The discriminatory power of the SDS measure in health inequality monitoring

Next, as Table 3 shows, the SDS measure appears to have an overall significant discriminatory power, irrespective of the cutoff being used, the country under analysis, or the health indicator under scrutiny. As expected, dividing the population into four subgroups (non-deprived, less deprived, deprived, and extremely deprived) according to the intensity intervals bounded by the three cutoff values reveals that greater socioeconomic deprivation is associated with lower coverage of skilled birth attendance and DTP3 immunisation and higher prevalence of stunting and mortality rates. These results are consistent both in countries with a higher proportion of the population that are disadvantaged (e.g., Niger) and in countries with low levels of disadvantage (e.g., Tajikistan), and with only partial exceptions for mortality rates in India, Niger, and Papua Guinea to otherwise linear patterns across health indicators and intensity intervals. These partial exceptions are also a function of the underlying distributions of overlapping deprivations and corresponding distributions of people per SDS intensity interval.

**Table 3:** RMNCH Indicators by socioeconomic deprivation status in six countries

Country	Indicator	Non-deprived (< 0.25)	Less deprived (0.25–0.5)	Deprived (0.5–0.75)	Extremely deprived ($\ge$ 0.75)
Haiti	**n**	**1,094**	**4,257**	**941**	**1,436**
	Skilled birth attendance (%)	73.2	43.7	25.2	11.6
	DTP’ immunisation (%)	77.8	56.9	45.6	35.0
	Stunting (%)	12.5	18.3	31.7	37.3
	Under-five mortality rate	64.2	77.2	95.1	101.6
India	**n**	**130,238**	**96,847**	**45,289**	**10,713**
	Skilled birth attendance (%)	91.6	76.0	61.2	49.5
	DTP’ immunisation (%)	84.0	76.6	67.1	55.0
	Stunting (%)	29.1	4’.9	53.4	56.8
	Under-five mortality rate	40.0	62.8	75.9	71.6
Niger	**n**	**798**	**2,341**	**2,961**	**7,172**
	Skilled birth attendance (%)	93.2	59.1	30.1	17.4
	DTP’ immunisation (%)	91.5	82.6	70.1	62.2
	Stunting (%)	17.9	39.0	44.3	45.8
	Under-five mortality rate	54.5	125.8	167.5	156.0
Pakistan	**n**	**6,109**	**4,207**	**1,481**	**1,424**
	Skilled birth attendance (%)	84.5	6’.4	51.6	42.0
	DTP’ immunisation (%)	88.4	68.7	56.3	49.8
	Stunting (%)	26.3	41.8	48.7	61.1
	Under-five mortality rate	66.7	73.2	94.3	95.6
Papua New Guinea	**n**	**1,491**	**5,512**	**1,377**	**2,194**
	Skilled birth attendance (%)	93.1	64.4	45.8	30.4
	DTP’ immunisation (%)	55.5	49.6	34.3	25.6
	Stunting (%)	20.7	42.8	50.6	50.9
	Under-five mortality rate	35.0	48.3	47.3	65.1
Tajikistan	**n**	**5484**	**963**	**5**^**a**^	**0**
	Skilled birth attendance (%)	95.2	92.7	-	-
	DTP’ immunisation (%)	86.5	90.2	-	-
	Stunting (%)	17.3	18.6	-	-
	Under-five mortality rate	32.8	35.6	-	-

The *n* represents the total sample of individuals living in households with children under 5

^a^In Tajikistan, there were no individuals who experience more than 75% of the weighted sum of deprivations. Therefore, there is no information related to their levels of coverage in the selected indicators. Given the sample size of 5, we do furthermore not present estimates for those deprived in at least 50% of the weighted SDS indicators

Importantly, this exercise also answered the question whether the SDS measure has appropriate discriminatory power in countries where few people fall within some of its absolutely defined gradient levels. This question is not trivial because the SDS measure does not divide each population into more or less equally sized subsets (as relative measures may do). That is, the discriminatory power of the SDS measure is directly linked to power of data analysis and the sample size of individuals who experience each absolute level of socioeconomic deprivation in a given country. In some countries, such as Tajikistan, it is therefore limited to lower deprivation levels, as use of the SDS measure suggests that there are virtually no (extremely) socioeconomically deprived individuals in Tajikistan. The evidence presented in Table 3 shows that the SDS measure was able to discriminate and uncover unequal distributions of health indicators across the possible spectrum of disadvantage levels in all countries and for all health indicators.

#### Exercise 2: Comparative analysis of the SDS measure and the DHS wealth index

A comparative analysis of the new SDS measure with the DHS Wealth Index showed that both measures are highly associated across all six study countries (Table 4). Indeed, when analysing both measures as categorical variables, individuals in the highest wealth index quintiles are predominantly found in the lowest SDS intensity interval across all six countries analysed. Both variables are significantly dependent in all countries according to the chi-squared test performed (*p*-values < 0.001). Results look similar when discrete quantitative variables, i.e., weighted deprivation scores and DHS Wealth Index values, are used for Pearson correlations.

**Table 4:** Percentage of people in DHS wealth index quintiles by socioeconomic deprivation status

Country	SDS (four subgroups)	Q1 (poorest)	Q2	Q3	Q4	Q5 (richest)	Chi2 test (*p*-value)	Pearson correlation (*p*-value)^1^
**Haiti**	Non-deprived (< 0.25)	0.0	0.2	9.7	34.3	69.5	< 0.001	-0.736 (< 0.001)
	Less deprived (0.25-0.5)	12.2	47.7	66.1	57.3	28.9		
	Deprived (0.5-0.75)	37.1	41.5	22.1	8.2	1.6		
	Extremely deprived (≥ 0.75)	50.6	10.9	2.1	0.1	0.0		
**India**	Non-deprived (< 0.25)	1.0	22.5	68.8	90.7	97.4	< 0.001	-0.791 (< 0.001)
	Less deprived (0.25-0.5)	48.8	58.7	28.1	8.8	2.5		
	Deprived (0.5-0.75)	40.7	17.4	3.0	0.5	0.7		
	Extremely deprived (≥ 0.75)	9.5	1.41	0.1	0.0	0.0		
**Niger**	Non-deprived (< 0.25)	0.0	0.0	0.0	0.0	30.1	< 0.001	-0.754 (< 0.001)
	Less deprived (0.25-0.5)	0.9	3.0	7.0	10.8	32.1		
	Deprived (0.5-0.75)	12.3	23.7	35.0	40.7	27.4		
	Extremely deprived (≥ 0.75)	86.8	73.2	57.9	48.5	10.3		
**Pakistan**	Non-deprived (< 0.25)	4.7	31.1	62.6	82.4	92.8	< 0.001	-0.768 (< 0.001)
	Less deprived (0.25-0.5)	22.1	38.1	29.3	15.9	6.9		
	Deprived (0.5-0.75)	39.8	24.8	7.7	1.8	0.3		
	Extremely deprived (≥ 0.75)	33.4	6.0	0.4	0.0	0.0		
**Papua New Guinea**	Non-deprived (< 0.25)	0.0	0.0	0.0	1.7	61.4	< 0.001	-0.754 (< 0.001)
	Less deprived (0.25-0.5)	5.1	20.3	40.6	66.4	31.7		
	Deprived (0.5-0.75)	35.5	43.2	42.4	27.3	6.4		
	Extremely deprived (≥ 0.75)	59.3	36.4	17.0	4.5	0.5		
**Tajikistan**^a^	Non-deprived (< 0.25)	76.7	85.3	88.2	89.5	94.0	< 0.001	-0.405 (< 0.001)
	Less deprived (0.25-0.5)	21.5	14.1	11.3	10.0	5.9		
	Deprived (0.5-0.75)	-	-	-	-	-		
	Extremely deprived (≥ 0.75)	-	-	-	-	-		

^a^In Tajikistan, there were no individuals who experience more than 75% of the weighted sum of deprivations. Given the sample size of 5, we do furthermore not present estimates for those deprived in at least 50% of the weighted SDS indicators

^1^This analysis was conducted using the DHS Wealth Index score, i.e. the first principal component, and the SDS counting vector

However, as expected when comparing an absolute (SDS) and a purely relative (DHS Wealth Index) measure, there also is considerable cross-country variation. In India, for example, about half (49.8%) of those in the bottom DHS wealth quintile experience less than 50% of the weighted SDS deprivations and thus fall within the bottom, less or non-deprived SDS categories. In Tajikistan, more than three in four people in the bottom wealth quintile (76.7%) are in the least disadvantaged SDS interval. This empirically testifies to the value-added of considering absolute disadvantage as per SDS in addition to relative, unidimensional disadvantage as per DHS Wealth Index, confirming their conceptual and analytical complementarity.

#### Exercise 3: Sensitivity and robustness across varying multidimensional cutoffs

Sensitivity and robustness checks for SDS measure results across a range of reasonable multidimensional cutoffs were performed on DHS data for a set of 46 countries. This analysis was aimed at understanding the robustness of countries’ rank order by the incidence of multidimensional disadvantage across varying cutoffs. A measure that is overly sensitive to reasonable changes in some parametric choices would, arguably, be somewhat problematic (see also Alkire et al. 2020, n.d.). Overall, however, the results of this exercise revealed that country orderings are largely maintained when cutoffs are changed. Indeed, the computation and analysis of 1035 pairwise comparisons found that across a range of cutoffs from 16.67%-91.67% of weighted SDS deprivations (weighted deprivation scores between 2/12 and 11/12 to be precise), 87% of the pairwise comparisons were significantly robust. Therefore, there were 909 identical rank-pairs across cutoffs. In addition, the performance of a Kendal Tau-b rank correlation revealed that in almost 80% of the analysed cases (again, for cutoff values between 16.67% and 91.67%), rank comparisons yielded concordant pairs. Thus, country orderings by the incidence of multidimensional disadvantage were maintained in most of the cases, irrespective of the multidimensional cutoff chosen in the range from 16.67%-91.67%.

#### Exercise 4: Disaggregating and breaking down the SDS measure

The final exercise illustrated some of the axiomatic properties of the SDS measure, namely subgroup decomposability and indicator-wise breakdown, which can be particularly salient when considering policy and programming entry points for tackling health inequities and key social determinants of health. This exercise used 2016 DHS data from Haiti and one overall deprivation cutoff at 50%. Thus, a person is multidimensionally deprived if they live in a household deprived in at least 50% of the weighted SDS deprivations. Disaggregation can be performed on all components of an Alkire-Foster measure (see also methods section above and [10, 11]). For illustrative purposes, this analysis entailed urban–rural disaggregation of the incidence and intensity of multidimensional disadvantage, as well as their product, the ${M}_{0}$, along with its composition for urban versus rural Haiti. The analysis of SDS composition builds on the dimensional breakdown axiom that allows us to analyse how much each indicator contributes to overall multidimensional disadvantage among mutually exclusive and collectively exhaustive (sub-)populations. Urban–rural disaggregation of results revealed that people living in rural Haiti are more frequently socioeconomically deprived (as per SDS at a cutoff of 50% of the weighted deprivations) than those in urban areas (with an incidence of 39.0% versus 6.7%). In addition, the intensity of multidimensional deprivation is almost eight percentage points higher in rural areas compared to urban areas (72.6% vs 65.1%). Finally, the ${M}_{0}$ in rural areas is equal to 0.283 compared to 0.043 in urban areas (Table 5).

**Table 5:** SDS Incidence, intensity, ${\mathrm{M}}_{0}$ and prevalence of RMNCH Indicators among the socioeconomically deprived at $50\mathrm{\%}$ by rural–urban area in Haiti, 2016

	**National**	**Rural**	**Urban**	**Sig**
Incidence	26.1%	39.0%	6.7%	***
Intensity	71.2%	72.6%	65.1%	***
M_0_	0.187	0.283	0.004	***
**For those multidimensionally deprived …**				
Skilled birth attendance	17.3%	15.1%	37.8%	***
DTP3 immunisation	39.4%	39.1%	42.2%	
Stunting	34.7%	34.9%	32.6%	
Under-five mortality rate	99.1	85.9	108.2	

^***^ *p*-value < 0.001

**Fig. 1:**
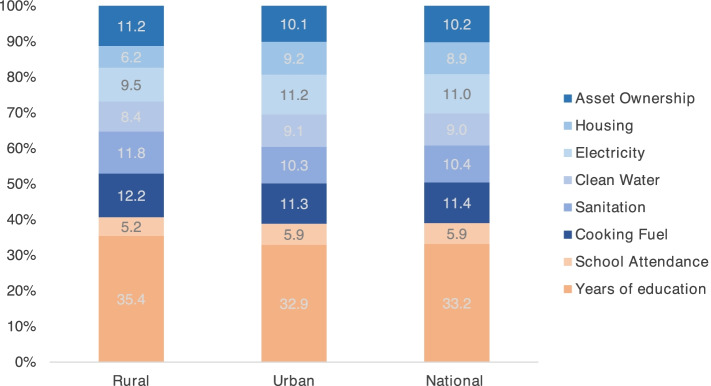
The relative contribution of each indicator to the SDS measure

An analysis of the contributions of each SDS indicator to the ${M}_{0}$ showed that the composition of socioeconomic deprivation is, despite the considerable difference in incidence, overall similar. Some differences were observable, though. Deprivation of basic assets, improved sanitation, clean cooking fuel, and a minimum year of education, for example, contributed more to ${M}_{0}$ in rural than in urban Haiti (Fig. 1). Such results, here presented for purely illustrative purposes, can be insightful to detect which deprivations drive overall multidimensional disadvantage in different populations or population subgroups, and thus help to develop well-targeted interventions to reduce the overall deprivation load and de-cluster disadvantages.

Disaggregated results also showed that the multidimensionally disadvantaged population (given the multidimensional cutoff of $50\%$) in rural Haiti included a significantly lower percentage of women whose deliveries were attended by skilled health personnel (15.1% in rural areas vs. 37.8% in urban areas), whilst there were no detectable, statistically significant urban–rural inequities for the multidimensionally deprived in the other three health indicators (Table 5).

## Discussions

Though further analyses will be required, the SDS measure has several advantages for health inequality monitoring and analysis efforts. First, there is widespread recognition that human development and disadvantage are multidimensional phenomena, in which dimensions compound and intersect, that can neither be sufficiently conceptualised nor measured through reliance on single, unidimensional indices or proxies such as material wealth or monetary means to ends. However, health inequality monitoring efforts commonly rely on exactly such unidimensional measures or proxies, e.g., income, wealth, or single indicators of educational attainment. To be potentially used as a complementary measure, the proposed SDS measure contributes to closing this gap in operationalising a multidimensional concept for monitoring purposes. Focused on people’s achievement or deprivations across two dimensions of human development (material living standard and education), it identifies as worse-off those who are simultaneously affected by multiple deprivations. Unlike other composite indices that use aggregate populations as unit of identification, the new measure works at the level of each household.

Second, some commonly used dimensions for global health inequality monitoring rely on relative measures of unidimensional deprivation. Such relative and endogenously defined measures do not allow for comparisons across time and space. In addition, relative measures do not capture notions of absolute welfare or disadvantage but rather remain implicit inequality metrics in themselves. There is thus demand for an appropriate multidimensional measure of absolute disadvantage to facilitate health inequality monitoring in line with these conceptual and ethical concerns. The proposed SDS measure operationalises exactly these concerns, independent of any given distribution and rather anchored in exogenously defined and normatively justified thresholds (see also [19]).

Third, these and additional concerns apply especially to some of the most commonly used indices to stratify health indicators, such as the DHS Wealth Index [3]. As other relative and purely endogenously derived measures, the DHS Wealth Index is not comparable across time and space, and neither can it be disaggregated or decomposed. The SDS, on the other hand, as an exogenously defined, absolute measure with a harmonised structure, allows for intertemporal and inter-spatial measurement and analysis. This applies, as illustrated above, for findings on the incidence, intensity, ${M}_{0}$*,* as well as the composition of multidimensional disadvantage – all of which can be compared across time and space, both internationally and by disaggregation for subgroups of the same population. Since such analyses are not usually possible with other measures, they present a clear value-add of the SDS measure. This can prove insightful when applied in future empirical studies and efforts to monitor and reduce health inequity around the world.

Key advantages of the SDS measure thus include its operationalisation of conceptual intuitions on absolute disadvantage; its household-level identification function; its ability to be disaggregated by mutually exclusive and collectively exhaustive population subgroups (e.g. subnational regions, urban–rural areas, gender of household head, etc., depending what the underlying data allow for), which allows for comparisons across groups; and that it can be used to uncover the composition of multidimensional disadvantage, making use of the dimensional breakdown property satisfied by the Alkire-Foster class of measures. Disaggregation and breakdown by indicator allow for policy-salient analyses of socioeconomic deprivation and health inequality alike that are not possible with wealth indices. Yet, importantly, the SDS measure is not proposed as a substitute for existing unidimensional indices such as money metrics, single educational indicators, or wealth indices. Rather, the discussion above explains the motivation for the construction and proposal to use the SDS measure complementary (i.e., in purposeful addition) to these and other measures for global health inequality monitoring efforts.

Other measures formally not dissimilar to the one proposed herein have also been previously used for similar purposes (see, i.e., [20–22]), so the presently derived measure and application is not entirely without motivation and precedence. It instead follows now commonly accepted notions of how to conceptualise and measure socioeconomic deprivation or disadvantage. However, these previous studies were mostly focused on individual countries and single health outcomes, whilst the SDS measure is harmonised for use in more than 100 countries around the world. Formally and parametrically, the work by the *Lancet Commission on NCDI Poverty* [23–25] used a multidimensional measure similar to the SDS for a joint analysis of non-communicable diseases and injuries among people in some of the multidimensionally most deprived countries. The SDS measure, however, is markedly different in two key parametric choices: the number and value of cutoffs it applies as well as the relative weights that each of its components receives.

Lastly, some limitations of the SDS measure and frontiers for future research are worth highlighting. The selection of the household as the unit of identification assumes that individual deprivations and achievements are equally shared between household members. Thus, it does not directly allow for straightforward and comprehensive analyses of intra-household inequalities (e.g. by age, gender, etc.), though these can be part of additional analytical exercises. This is a clear shortcoming that is entirely driven by data limitations. It is, however, also not at all a unique feature of the new measure. Household-level identification is indeed also applied in measures such as the DHS wealth index, as well as of most other measures of unidimensional (i.e., monetary metrics) and multidimensional (e.g., multidimensional indices of poverty or welfare) (dis)advantage. In addition, finding and maintaining a global standard for the measurement of absolute deprivation is non-trivial. Our analyses showed that the discriminatory power of the SDS measure is, expectedly, limited where it is restricted to fewer gradient intervals. This was the case in Tajikistan, where few people were identified as more intensely socioeconomically deprived. Further analyses on low deprivation countries will be important, and if absolute socio-economic deprivation does generally decrease over time internationally, changes in the structure of the SDS measure may become necessary to adequately capture and discriminate absolute multidimensional disadvantage and associated inequalities in health. Similarly, there are other important dimensions and indictors of deprivation currently not included in the measure due to data limitations. Social and political inclusion and participation, decent work, freedom from discrimination, and other important indicators could be included in measures such as the SDS in the future, if appropriate and comparable data are standardly collected as part of international surveying efforts. The SDS measure and its underlying methodology would allow for much more empirical analyses than this introductory and methodological paper has covered.

Overall, results profiled suggest that a more comprehensive analysis of a larger set of countries and indicators could offer further valuable empirical insights. The measure can equally be applied to other datasets and variables than the ones studied herein. Expanding the set of health indicators, countries, and datasets analysed thus is an important next step to further ascertain the added value that the SDS measure could potentially provide for global efforts in health inequality monitoring. This can also be done with analyses of changes over time, for which the harmonised and exogenously defined structure of the SDS allows. This article did not analyse the characteristics of groups with higher levels of disadvantage (e.g., by age, subnational region, or ethnic identity – to name just a few) or the composition of the disadvantage in each country (i.e., identifying which indicator contributes the most). These points, too, outline important questions for future research.

## Conclusion

This article presented the Socioeconomic Deprivation Status (SDS) measure to monitor health inequalities and illustratively applied it to a set of countries and indicators included in the *WHO Health Equity Monitor* database. We analysed the discriminatory power of the measure across four RMNCH indicators and tested it using data from six countries with different levels of disadvantage and economic development. With the above-mentioned partial exceptions of Under-five mortality rates in India, Niger, and Papua New Guinea, the higher the level of disadvantage, the lower the level of achievement in each of the four health indicators. These results were consistent across all six countries analysed. We found that no matter the level of disadvantage, the SDS measure shows important differences between groups. Additionality, we compared the new SDS measure with the DHS Wealth Index in the six countries. Results showed that both measures presented coherent results. The intensity of disadvantage is negatively associated with the values of the DHS Wealth Index. This correlation was significant for all countries studied. However, the comparative analysis also showed considerable variation in the agreement between both measures across countries, confirming the complementarity of relative DHS Wealth Index quintiles and absolute SDS measure gradient. Finally, we illustrated some of the SDS measure’s axiomatic properties using data from Haiti. Disaggregating the measure by rural and urban areas, we found that the levels of multidimensional deprivation are higher in rural areas. Concludingly, this initial exploration pointed to important areas for further research, but also identified that the SDS measure has several properties that make it, in principle, of salience for applications to health inequality measurement efforts around the world.

## Supplementary Information

**Additional file 1.**

## Data Availability

The datasets used for this study are available in the DHS Program website.

## Abbreviations

DHS: Demographic and Health Surveys
DTP3: Three doses of the combined diphtheria, tetanus toxoid and pertussis vaccine
NCDI: Non-communicable diseases and injuries
OPHI: Oxford Poverty & Human Development Initiative
RMNCH: Reproductive, maternal, neonatal, and child health
SDS: Socioeconomic deprivation status
U5MR: Under-five mortality rate
UNDP: United Nations Development Programme
WHO: World Health Organization
