# Correction: Exploring the potential for a new measure of socioeconomic deprivation status to monitor health inequality

**DOI:** 10.1186/s12939-022-01696-3

**Published:** 2022-07-19

**Authors:** Jakob Dirksen, Monica Pinilla-Roncancio, Fernando C. Wehrmeister, Leonardo Z. Ferreira, Luis Paulo Vidaletti, Katherine Kirkby, Theadora Swift Koller, Anne Schlotheuber, Heriberto Tapia, Cecilia Vidal Fuertes, Sabina Alkire, Aluisio J. D. Barros, Ahmad Reza Hosseinpoor

**Affiliations:** 1 Oxford Poverty and Human Development Initiative, University of Oxford, Oxford, UK; 2 Universidad de los Andes, Bogotá, Colombia; 3 International Center for Equity in Health, Post-Graduate Program in Epidemiology, Federal University of Pelotas, Pelotas, Brazil; 4 World Health Organization, Geneva, Switzerland; 5 Human Development Report Office, United Nations Development Programme, New York, USA

**Correction: Int J Equity Health 21, 56 (2022)**

**https://doi.org/10.1186/s12939-022-01661-0**

After publication of this article [1], the authors reported that in several places in Table 3, a single quote (‘) occurs instead of the digit “3”.

It was also reported that the copyright holder for this article was incorrectly given as 'The Author(s)', but should have been 'WHO'.

The original article [1] has been updated.
