# Supplementary material for: Exploring the potential for a new measure of socioeconomic deprivation status to monitor health inequality
Source: Int J Equity Health. 2022 Apr 23;21:56. doi: 10.1186/s12939-022-01661-0 (PMC9034442; doi:10.1186/s12939-022-01661-0)
Supplement: Supplementary file 1 — Additional file 1. [file 12939_2022_1661_MOESM1_ESM.docx]

# Appendix

Table A1. Overview of DHS Datasets Used

| **Country** | **Year** |
| --- | --- |
| Afghanistan | 2015-2016 |
| Albania | 2017-2018 |
| Angola | 2015-2016 |
| Armenia | 2015-2016 |
| Benin | 2017-2018 |
| Burkina Faso | 2010 |
| Burundi | 2016-2017 |
| Cambodia | 2014 |
| Chad | 2014-2015 |
| Colombia | 2015-2016 |
| Comoros | 2012 |
| Egypt | 2014 |
| Ethiopia | 2016 |
| Gabon | 2012 |
| Ghana | 2014 |
| Guatemala | 2014-2015 |
| Guinea | 2018 |
| Haiti | 2016-2017 |
| Honduras | 2011-2012 |
| India | 2015-2016 |
| Indonesia | 2017 |
| Jordan | 2017-2018 |
| Kenya | 2014 |
| Liberia | 2013 |
| Malawi | 2015-2016 |
| Maldives | 2016-2017 |
| Mali | 2018 |
| Mozambique | 2011 |
| Myanmar | 2015-2016 |
| Namibia | 2013 |
| Nepal | 2016 |
| Nicaragua | 2011-2012 |
| Niger | 2012 |
| Nigeria | 2018 |
| Pakistan | 2017-2018 |
| Papua New Guinea | 2016-2018 |
| Philippines | 2017 |
| Rwanda | 2014-2015 |
| Senegal | 2017 |
| South Africa | 2016 |
| Tajikistan | 2017 |
| Tanzania | 2015-2016 |
| Timor-Leste | 2016 |
| Uganda | 2016 |
| Yemen | 2013 |
| Zambia | 2018 |
